# Evaluating the Sputum Bacteriological Profile of Lower Respiratory Tract Infections With Bartlett Score Analysis in a Tertiary Care Hospital in Southern Karnataka

**DOI:** 10.7759/cureus.61924

**Published:** 2024-06-07

**Authors:** Rashmi P Mahale, Param Darpan Sheth, Neetha S Murthy

**Affiliations:** 1 Clinical Microbiology, Jagadguru Sri Shivarathreeshwara (JSS) Medical College and Hospital, Mysore, IND; 2 Microbiology, Jagadguru Sri Shivarathreeshwara (JSS) Medical College and Hospital, Mysore, IND

**Keywords:** bartlett score, antibiogram, bacterial profile, sputum, lower respiratory tract infection

## Abstract

Introduction: Lower respiratory tract infections (LRTIs) are one of the most commonly encountered infections with significant mortality and morbidity. Sputum is the most frequently obtained sample for LRTI diagnosis. However, sputum samples carry the risk of being non-representative due to the risk of contamination with oral colonizers. To overcome the dilemma with respect to representative sampling, the use of a scoring system such as the Bartlett scoring system is emphasized. This study probes the bacterial profile of sputum samples among patients presenting with LRTIs and their antibiotic susceptibility profile in relation to the Bartlett scoring system.

Methodology: Retrospective data for a period of three years, comprising 4960 sputum samples from patients presenting with LRTI, were collected to study the bacterial profile and antibiogram in comparison with the sputum quality analyzed by the Bartlett scoring system.

Results: Out of the 4960 sputum samples analyzed from patients with LRTI, 31.18% yielded the growth of bacterial pathogens, and 98.64% of the sputum samples yielding pathogenic growth had a significant Bartlett score.

Conclusion: Sputum samples are non-invasive representative samples of lower airway infective pathologies. Sputum quality assessment by Bartlett scoring serves as a proxy marker to rule out respiratory colonization and aid culture-based diagnosis.

## Introduction

Lower Respiratory Tract Infection (LRTI) is an umbrella term used for a wide variety of diseases affecting the tertiary bronchioles and the lung. Diseases classified under LRTI are pneumonia, acute exacerbations of chronic obstructive pulmonary disease/chronic bronchitis, and acute exacerbation of bronchiectasis [1].

In clinical practice, LRTIs are the most encountered conditions both in the community and hospital settings [2]. According to the study conducted on the Global Burden of Disease in 2019, LRTIs were the 4th leading cause of disability-adjusted life years as well as health loss globally for both sexes combined [3]. Of these LRTIs, Pneumonia incidence is unprecedented and is one of the most important LRTIs. India accounts for about 23 percent of global pneumonia cases seen in children under the age group of five years, and the reported mortality rate ranges anywhere from 14% to 30% [4]. In the year 2015, the burden of pneumonia among immunocompetent children under five years of age was found to be 40.3% [5]. Pneumonia case fatality rates vary according to healthcare settings, geographical region, patient categories, and age across various states of India [6].

Sputum is the most easily accessible respiratory specimen collected for isolating infectious agents and diagnosing LRTIs. The accuracy of isolating the infectious agent depends on the quality of the sample sent for investigation. Emphasis on accurate specimen collection is indispensable to achieve accurate identification of the etiological agent. Sputum, though representing the lower respiratory tract, may be contaminated with oropharyngeal flora. The dilemma of whether the isolated bacteria from the sputum sample is a true pathogen or a mere colonizer of the upper respiratory tract exists. To overcome this, various scoring systems are used to assess the quality of sputum, such as Bartlett, Murray and Washington [7-9], where the quality of the sputum is assessed by the presence of squamous epithelial cells and inflammatory cells observed under the low power of the microscope.

In the setting of healthcare, antibiotic overprescribing is not a new issue but rather a rampant one, senseless disposition of antibiotics leads to the development of strains of micro-organisms that are resistant to these antibiotics, thereby making them immune to conventional therapy. This study aims to detect any change in the bacteriological profile of sputum samples and their antibiotic sensitivity pattern in co-ordination with the importance of scoring system to assess the quality of sputum samples for the management of LRTIs.

## Materials and methods

Study design and duration

This is a retrospective, single-center hospital-based observational study carried out at Jagadguru Sri Shivarathreeshwara (JSS) Medical College and Hospital, Mysore, Karnataka, India. Data was analyzed for a period of three years from 1/1/2020 to 31/12/2022. The study was conducted retrospectively to analyze the Bartlett score against the bacteriological profile of sputum samples and their susceptibility pattern over a period of three years analyzed by the VITEK 2 Compact system (Biomerieux, New Delhi, India) in accordance with Clinical Laboratory Standards Institute (CLSI) guidelines [10].

Study subjects

All hospital patients, either from the OPD (Outpatient Department) or wards from all departments of the hospital, who fulfill the inclusion criteria were included in the study.

Inclusion and exclusion criteria

All patients of age >=18 years, presenting with cough and sputum production, temperature above 37.8 degrees Celsius, WBC count of greater than 12,000 cells/μl, pleuritic chest pain or dyspnea irrespective of co-morbidities were included in the study. The sputum samples from these patients were subjected to culture and susceptibility testing after scoring them by Bartlett’s scoring system [11]. Patients who were infected with HIV (human Immuno-deficiency virus) or immunocompromised or suffering from bone marrow suppression, and samples other than sputum samples were excluded from the study.

Sample collection

Spontaneously expectorated sputum samples, which were collected in sterile screw-capped containers as per routine laboratory protocol and sent to the microbiology laboratory at room temperature immediately within one hour of sample collection were included as the sample pool in this study. 

Methodology

The sputum samples provided by the patients and processed in the Department of Microbiology of the tertiary care hospital were retrieved retrospectively over a period of three years. The retrospective data set included Bartlett scores of the sputum samples given on the basis of gram stain findings. As per the Bartlett scoring system, sputum samples were graded on a numerical scale from -2 to +3 as depicted in Table 1. Grading was based on (1) the number of neutrophils per low-power field, (2) the presence of mucous strands, (3) the number of squamous epithelial cells per low-power field [11].

**Table 1:** Bartlett scoring system [11] LPF: Low-power field

Number of neutrophils/10x LPF	Grade
<10	0
10 to 25	+1
>25	+2
Presence of Mucus	+1
Number of epithelial cells/10x LPF	Grade
10-25	-1
>25	-2
Total score	

Culture and antibiotic susceptibility test results performed as per the laboratory protocols in agreement with the National Accreditation Board for Laboratories (NABL) standards by automated identification and antibiotic susceptibility testing using the VITEK-2 compact system were retrieved [10]. Clinical and demographic data was fetched through the Hospital Information system. All data was analyzed using Microsoft Excel. The data set has been presented as numbers and percentages for the sake of comparative analysis.

## Results

Gram stain, Bartlett scoring and culture yield of the sputum samples

**Figure 1:**
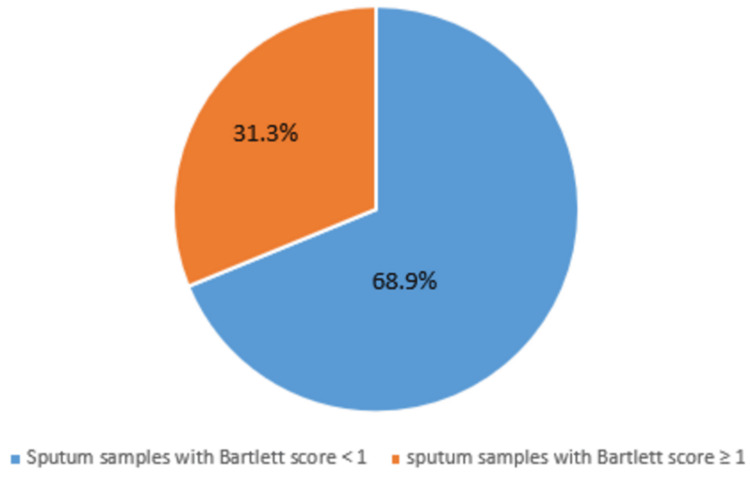
Graph demonstrating the Bartlett score of the tested sputum samples

A total of 4960 sputum samples were received from patients suspected of LRTI during the study period, of which 1547 (31.18%) samples yielded growth of pathogenic organisms. Of the 3413 samples that did not yield growth of pathogenic organisms, 245 had a Bartlett score of ‘-1’ and 2856 samples had a Bartlett score of ‘0’ and 312 samples had a Bartlett score of between +1 and +2 (Figure 1).

**Figure 2:**
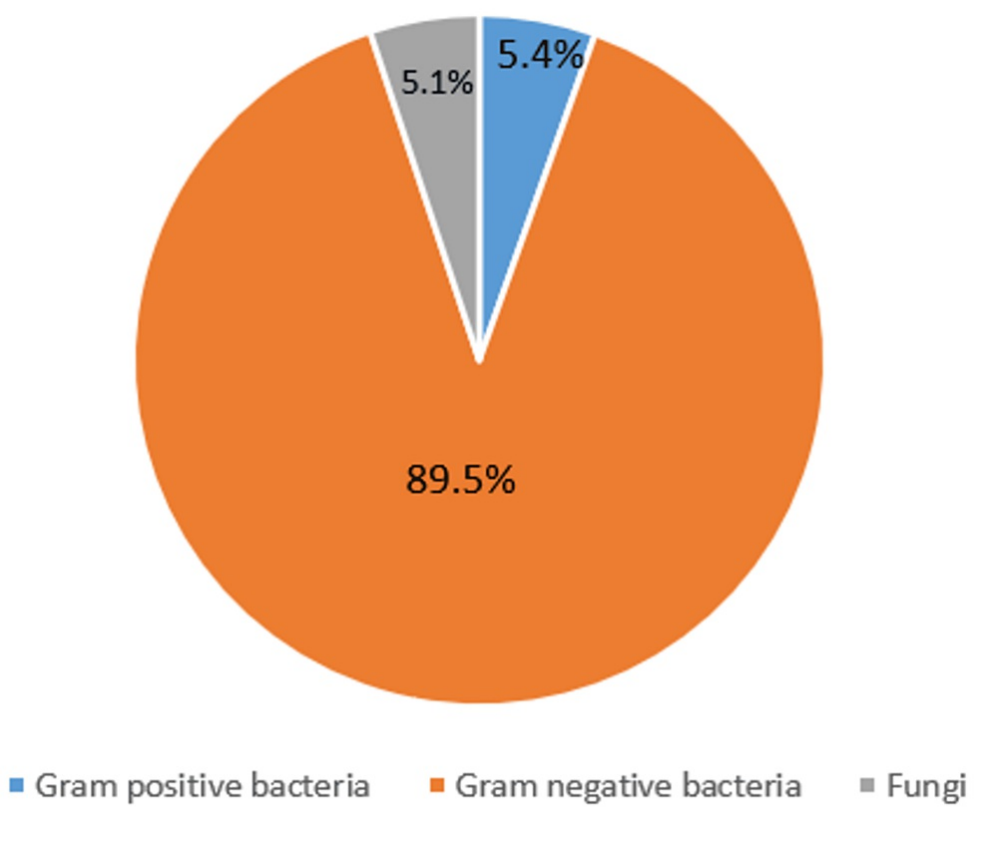
Graph demonstrating the distribution of sputum samples yielding bacteria and fungi Percentage of sputum samples that yielded growth of either Gram-positive bacteria, Gram-negative bacteria and fungi.

Among the samples that yielded growth of pathogenic organisms, 21 had a Bartlett score of ‘0’, and the remaining 1526 samples had a Bartlett score ranging from +1 to + 2 and were considered satisfactory samples. Among the 1547 (31.18%) samples that yielded growth, 1384 samples (89.46%) yielded Gram-negative bacteria, 84 samples (5.42%) yielded Gram-positive pathogens, and 79 samples (5.10%) yielded fungal pathogens (Figure 2).

Agewise distribution of samples

Age and gender-wise distribution of culture-positive samples. Samples from patients between the ages of 61 and 70 years was the most common (27.34%) age group yielding a positive sputum culture, followed by 52-60 years (21.65%). A male predominance (69.74%) was observed, as depicted in Table 2.

**Table 2:** Age-wise breakdown of LRTI cases LRTI: Lower Respiratory Tract Infection

Age group	Male	%	Female	%	Total	%
<10 years	9	0.58%	4	0.25%	13	0.84%
10-20 years	18	1.16%	15	0.96%	33	2.13%
21-30 years	32	0.20%	29	1.87%	61	3.94%
31-40 years	102	6.59%	41	2.65%	143	9.24%
41-50 years	163	10.53%	57	3.68%	220	14.22%
51-60 years	225	14.54%	110	7.11%	335	21.65%
61-70 years	294	19.00%	129	8.33%	423	27.34%
71-80 years	177	11.41%	59	3.81%	236	15.25%
81-90 years	51	3.29%	23	1.48%	74	4.78%
>90 years	8	0.51%	1	0.06%	9	0.58%
Total	1079	69.74%	468	30.25%	1547	99.71%

Distribution of Gram-positive bacteria

A total of 84 Gram-positive cocci were isolated, accounting for 5.42%. Of the Gram-positive cocci isolated, *Staphylococcus aureus* was the most common isolate, followed by *Streptococcus* species. *Pneumococci* accounted for 13.09% of Gram-positive isolates and was identified as the etiological agent of LRTI in only 0.71% of cases (Table 3).

**Table 3:** Distribution of various Gram-Positive bacterial isolates responsible for LRTIs LRTI: Lower Respiratory Tract Infection

Organism	Isolate n (%)
Staphylococcus aureus	33(39.28%)
Group A streptococcus species	40 (47.61%)
Pneumococci	11(13.09%)
Total	84

Distribution of Gram-negative bacteria

Gram-negative bacilli were the major pathogens responsible for LRTI in the present study. A total of 1384 Gram-negative bacilli were isolated, accounting for 89.46%. *Klebsiella* spp (47.18%) was the most common pathogen followed by *Pseudomonas* (21.31%) and *Acinetobacter* spp (17.55%) as depicted in Table 4.

**Table 4:** Distribution of various Gram-Negative bacterial isolates responsible for LRTI LRTI: Lower Respiratory Tract Infection

Organism	Isolate number
Klebsiella species	653
Pseudomonas species	295
Acinetobacter species	243
Escherichia coli	93
Enterobacter species	52
Serratia species	11
Stenotrophomonas maltophilia	10
Haemophilus influenzae	9
Citrobacter species	6
Sphingomonas paucimobilis	5
Burkholderia capceria	2
Morganella morganii	2
Provendicia rettgeri	1
Aeromonas salmonicida	1
Bordatella bronchiseptica	1
Total	1384

Antibiotic sensitivity pattern of Gram-positive cocci

Gram-positive cocci showed 100% sensitivity to vancomycin and linezolid and reduced susceptibility to erythromycin as enlisted in Table 5.

**Table 5:** The antibiotic susceptibility observed amongst Gram-positive bacteria causing LRTI LRTI: Lower Respiratory Tract Infections; S: Sensitive; R: Resistant; NA: Not Applicable

Antibiotic	Staphylococcus aureus (33), n (%)		Streptococcus species (40), n (%)		Pneumococci (11), n (%)	
	S	R	S	R	S	R
Erythromycin	9(27%)	24(73%)	14(43%)	18(57%)	8(72%)	3(28%)
Daptomycin	26(78%)	7(22%)	NA	NA	NA	NA
Levofloxacin	6(18%)	27(82%)	25(78%)	7(22%)	11(100%)	0(0%)
Vancomycin	33(100%)	0(0 %)	32(100%)	0(0%)	11(100%)	0(0%)
Linezolid	33(100%)	0(0%)	32(100%)	0(0%)	11(100%)	0(0%)
Gentamicin	24(72%)	9(28%)	NA	NA	11(100%)	0(0%)
Tetracycline	28(84%)	5(16%)	26(81%)	6(19%)	9(81.81%)	2(18.18%)
Ciprofloxacin	7(21%)	26(79%)	31(96%)	1(4%)	10(90%)	1(9%)
Trimethoprim/ Sulfamethoxazole	27(81%)	6(19%)	31(96%)	1(4%)	9(81.81%)	2(18.18%)
Clindamycin	18(54%)	15(46%)	27(87%)	5(13%)	10(90%)	1(9%)

Antibiotic sensitivity found in Gram-negative bacilli

Gram-negative isolates showed an increasing trend of resistance towards Beta-lactam and Beta-lactamase combinations, and cephalosporins, as depicted in Table 6.

**Table 6:** The antibiotic susceptibility observed amongst Gram-negative bacteria causing LRTI LRTI: Lower Respiratory Tract Infections; S: Sensitive; R: Resistant; NA: Not Applicable

Antibiotic	Klebsiella species, n(%)653		Pseudomonas species, n(%)295		Acinetobacter species, n(%)243	
	S	R	S	R	S	R
Amoxicillin/clavulanic acid	432(66%)	221(34%)	NA	NA	223(91%)	20(9%)
Piperacillin/ tazobactam	416(63%)	237(37%)	138(46%)	157(54%)	219(90%)	24(10%)
Cefuroxime	254(38%)	399(62%)	NA	NA	NA	NA
Cefuroxime axetil	261(39%)	392(61%)	NA	NA	NA	NA
Cefaperazone / sulbactum	453(69%)	200(31%)	164(55%)	131(45%)	221(89%)	22 (11%)
Cefipime	362(55%)	291(45%)	146(49%)	149(51%)	217(89%)	26 (11%)
Ceftriaxone	252(38%)	401(62%)	NA	NA	242(99%)	1(1%)
Imipenem	494(75%)	159(25%)	137(46%)	158(54%)	199(81%)	44(19%)
Meropenem	465(71%)	188(29%)	134(55%)	161(45%)	211(86%)	32(14%)
Ertapenem	495(75%)	158(25%)	294(99%)	1(1%)	242(99%)	1(1%)
Amikacin	590(90%)	63(10%)	228(77%)	67(23%)	216(88%)	27(12%)
Gentamicin	483(73%)	170(27%)	169(57%)	126(43%)	211(86%)	32(14%)
Ciprofloxacin	324(49%)	329(51%)	137(46%)	158(54%)	201(82%)	42(18%)
Tigecycline	631(96%)	22(4%)	NA	NA	153(62%)	90(38%)
Trimethoprim sulfamethoxazole	416(63%)	237(63%)	NA	NA	231(95%)	5(5%)

Multi-drug resistant organisms causing LRTI

Methicillin-resistance was seen in 51.5% of the *Staphylococcus aureus *isolates. Among the Gram-negative bacilli, 16.54% of isolates were found to be multidrug resistant.

## Discussion

In the present study, a total of 4960 sputum samples were received for culture and sensitivity, and all the samples were scored using Bartlett’s scoring system. Among these, 3413 (68.8%) samples had a score of ‘0’ or ‘-1’ and yielded no pathogenic bacteria. A similarly high rate (85.6%) of poor-quality sputum samples was reported by Popova et al. [12]. Among the 1547 sputum samples that yielded the growth of pathogenic organisms, 312 samples had a Bartlett score of ‘1+’/2+ but did not yield the growth of any pathogen. The cause of LRTI in these cases may be either attributed to viral etiology or partial antibiotic therapy. Of the samples that yielded the growth of pathogens, 21 samples were of poor quality but yielded a pure growth of a known respiratory pathogen like *Pneumococci, Klebsiella* spp., and *Streptococcus* species, and thus were reported.

The scoring system used in this study was Bartlett’s scoring system, in which sputum quality is assessed by the White blood cell-squamous epithelial cell ratio. Thus, to achieve accurate isolation of an etiological agent, there should be an emphasis on sample collection.

In the present study, a male predominance (69.74%) in LRTI was noted; this could be due to risk factors like smoking and alcoholism, which are more common in males. In the present study, the 60-70 years age group was found to be the most vulnerable age group (27.3%) for LRTI; this can be attributed to the unaccounted presence of other co-morbidities like diabetes, hypertension, COPD, etc., in this age group.

The overall sputum culture positivity was seen only in 31.1% of the samples; however, a slightly higher rate (49.3%) of positivity was observed in a study conducted by Khan et al. [13] and Santella et al. [14] who have reported (39.1%) of culture positivity. The low rate of culture positivity in the present study is attributed to the low quality of the sputum samples received and probably due to the use of antibiotics prior to sample collection. Gram-negative bacilli accounted for 89.1% of the pathogens, and Gram-positive was associated with only 5.42% of LRTIs. The most common pathogen associated with LRTI in the present study was *Klebsiella* species followed by *Pseudomonas* species and *Acinetobacter *species.* Pneumococci* accounted for only 0.71% of LRTI. A study from North America reported *S. pneumoniae* to be the most common pathogen (60%) followed by *Hemophilus influenzae*, in 1995 [9], thus depicting a change in the spectrum of bacteria causing LRTI. It is alarming to notice that in the present study, 17.6% of the etiological agents of LRTI were multi-drug resistant organisms (MDRO). Popova et al. have reported 14.5% of their isolates to be MDRO [12]. In the present study, methicillin-resistant *Staphylococcus aureus* (MRSA) contributed to 1.09%, MDR *Klebsiella* species accounted for 8.92%, *Acinetobacter* species 6.33%, MDR *Pseudomonas *species to 1.26% and MDR *E. coli* to 8.60% of MDR isolates. Five* E. coli*, eight *MRSA*, six *Acinetobacter *spp. and, 38 fungal isolates were isolated from poor-quality sputum samples, thus were of doubtful pathogenicity. Reporting without scoring or ignoring the scoring system results in the prescription of higher antibiotics and antifungals in patients, which would, in turn, result in the misuse of antibiotics further increasing the resistance among microorganisms. 

Study limitations

The study was of a retrospective nature and, hence, prone to possible confounding. The study determined the association of good-quality specimens with bacterial culture positivity. However, this study did not ascertain the presence of other possible fungal spectrum, viral, and parasitic respiratory pathogens in the sputum specimens. Prior antimicrobial therapy influencing the culture results has not been considered in the present study. This could be an added limitation of the study.

## Conclusions

Although sputum is an easily accessible specimen for diagnosing respiratory pathogens it is subject to contamination by oral commensals. The present study stresses the importance of good-quality sputum specimens graded in accordance with a scoring system. A poor-quality sputum specimen sent for culture and sensitivity yielding the growth of an oral commensal on the laboratory culture plate may misguide the clinician thereby resulting in misuse of antibiotics. Antibiotic misuse heightens the prevalence of multidrug-resistant pathogens. To break this vicious cycle, it is of utmost importance to assess the quality of the sputum sample before mechanical laboratory processing earmarking the importance of laboratory stewardship to combat Antibiotic resistance.
